# Radioactive Seed Localization for Conservative Surgery of Nonpalpable Breast Cancer: Recommendations for Technology Implantation Program

**DOI:** 10.29337/ijsp.182

**Published:** 2022-11-15

**Authors:** Hortência H. J. Ferreira, Carla Daruich de Souza, Maria Elisa C. M. Rostelato

**Affiliations:** 1 Nuclear and Energy Research Institute, IPEN, 2242 Prof. Lineu Prestes Avenue – Butantã, São Paulo, Brazil

**Keywords:** Breast cancer surgery, Impalpable breast lesion, Radioactive seed localization

## Abstract

**Background:**

The radioactive seed localization (RSL) is used in impalpable breast cancer conservative surgery to assist the surgeon in accurately locating and excising the lesion site. This study aims to present recommendations about the RSL program implementation in health institutions that perform breast cancer conservative surgery with intraoperative localization.

**Methods:**

An extensive literature review was performed. It comprehends: the committee responsible for implementation of the program actions; description of the necessary multidisciplinary team; the radiological safety committee role; the facility licensing; professionals training; material and instrumentation associated with the technique; and seed tracking system.

**Results:**

13 topics are presented. The **Program Implementation Committee** must be formed by leaders from each department. The committee assumes responsibility for evaluating the necessary processes and presenting the schedule for program implementation. Since the procedure is classified as a nuclear medicine procedure it requires **licensing**. The **Professional Team Formation, Education, and Training** is a priority and simulation exercises are necessary. The **Materials and Instrumentation Associated with the Technique** must be well-know by the team and they should practice using radiation detectors. The seed must be always tracked, from moment they are received to discard. An **Inventory for Tracking Seeds** is provided. The **Radiological Safety Aspects** such as the ALARA principle are presented. A full description for the **Radiological Procedure for Placing the seeds, the surgical removal** and the **Specimen Handling in Pathology** focusing on how to locate the seed and retrieve them. After removed, the seeds can be placed in storage to wait for full radioactive decay or be returned to the manufacturer.

**Conclusions:**

The procedure has the advantage to increase to 2 months the time between insertion of the seed and the surgical removal. Regular multidisciplinary team meetings during program development are important to create a realistic timeline, having briefing meetings after the first 1-5 RSL cases and having annual or biannual follow-up meetings to discuss any issues or incidents.

**Abstract Graphic Image:**
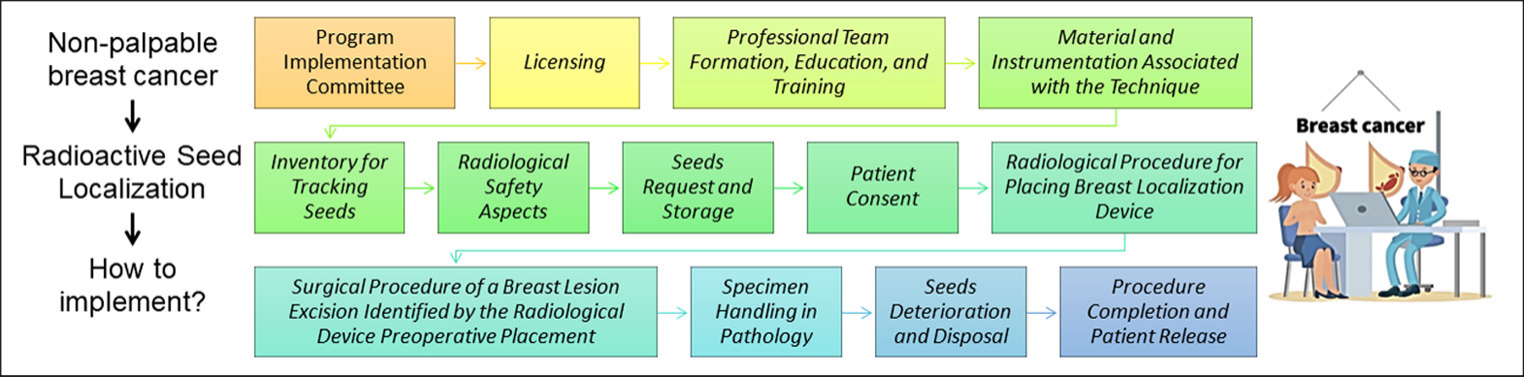
Created by Macrovector, obtained in Freepik at https://br.freepik.com/fotos-vetores-gratis/oncologia.

**Highlights:**

- This study present recommendations for RSL program implementation in hospitals
- Was performed by an extensive descriptive and qualitative literature review
- Topics 1: Implementation Committee, Professional Team Training, Instrumentation
- Topics 2: Radiological Safety, Patient Consent, Radiological Process
- Topics 3: Surgical Procedure, Pathology, Seeds Disposal Completion

## 1. Introduction

With the advancements in cancer detection, breast cancers are being diagnosed sooner. Small, non-palpable lesions present no visible change when compared to normal tissue making it difficult for the physician to locate the target area during a removal surgery.

The radioactive seed localization (RSL) is an intraoperative localization technique used in breast cancer conservative surgery. The technique uses a radioactive seed placed in the center of the target up to 60 days prior to removal surgery. When the removal occurs, the physician uses a radiation detector to locate and remove the seed and surrounding tissue [1].

RSL is a relatively new technology, the first reports of its use occurred in 1999 [2]. Pavlicek [1] was one of the pioneers to study the topic. In 2006 he published a survey that aid in the stablishment of guidelines to guarantee the safe use of Iodine-125 sources as a marker for locating non-palpable breast lesions in clinical routine. In 2019, Reed et. al. [3] carried out a study aimed at calculating the absorbed dose in residual breast tissue after RSL, via simulation in the Matlab software (MATrix LABoratory), based on clinical data from 262 patients. A seed with 2.00 MBq of activity was placed in a 1.00 mm lesion for 19.2 hours delivering average of 0.50 mGy. Several authors [4,5,6] report that RSL shows promising results in diminishing surgical margins, reoperation, and disease recurrence when compared to the wire-guided localization (WGL) technique, which is the main procedure currently used for this purpose.

To start RSL implementation several steps are necessary, such as the procedure committee, staff education (radiology, surgery, pathology, radiological safety, breast imaging, and nuclear medicine), and the radiological safety committee, and approval committee to investigate and comply to federal and state-specific nuclear regulatory standards. Also, the material and instrumentation inventory associated with the technique and seed tracking system, include emergency recovery procedures, are necessary.

RSL program will require an institution-specific Standard Operating Procedure protocol, which should establish the professional practices related to radiation safety aspects, the request and storing of seeds, the patient consent, the radiological procedure for placing breast localization devices, the surgical procedure, post-surgical pathology, seed disposal, and patient release.

This study aims to present recommendations for RSL program implementation in health institutions.

## 2. Methodology

This study was performed by an extensive descriptive and qualitative literature review. Searches were carried out in the Embase, PubMed, and Web of Science databases, using the following descriptors and keywords: nonpalpable breast cancer, breast conservative surgery, and radioactive seed localization.

Studies such as reports and practice protocols were selected, which provided considerations about the implementation of RSL technology in a health institution for the surgical localization of impalpable breast tumors. The selected studies went through a process of data ordering and final analysis. The ordering corresponds to the mapping and extraction of the data found, which was directed to collect data on pre-implementation requirements of RSL technology, as well as the protocols that must be part of the standard operating procedure of an RSL program. In turn, the final analysis concerns the realization of articulations between the data and the theoretical references, which allows producing a qualitative data synthesis.

### 2.1 Iodine-125

Iodine-125 is produced in a nuclear reactor by activation of Xenon-125. After decay by electron capture, it converts internally into Tellurium-125 emitting in the process photons of 27 keV, 31 keV and 35 keV, with an average energy of 29 keV. It has a half-life of 59.4 days [7]. Next the decay equation is shown.


{Xe^{125}}{\overset{{T_1/2}=16.9h}{\rightarrow}} {I^{125}}{\mkern 1mu} {\overset{{T_1/2}=59.4d}{\rightarrow}} T{e^{125}}

Iodine-125 emits photons with 27.5, 27.2, 30.1, and 35.5 keV (average of 29 keV). Due to the low average emission energy, its photons have little penetrating power [8,9].

### 2.2 Production of Iodine-125 seed

Iodine-125 seed is manufactured by several companies in the world such as GE Healthcare [10,11], Best Medical [12,13,14], and Eckert & Ziegler [11]. The seed consists of:

- A core that contains iodine-125 and a radiological marker, which can be gold, silver, ceramic, resin, etc.;
- A capsule of biocompatible material that surrounds the core, most commonly titanium or stainless steel [15].

**Figure 1:**
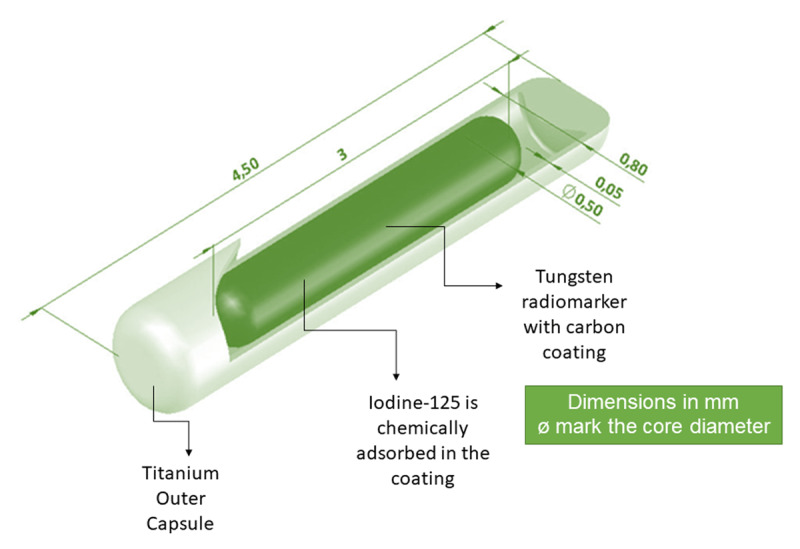
Example of Iodine-125 from Best Medical, model 2301.

An example of the Best Medical seed is shown in Figure 1.

Depending to the treatment that the seed is being used, the seed activity will vary. For example, to treat prostate cancer Iodine-125 seed with activities averaging 30 MBq [15]. For use in RSL, several authors report activities between 1.10 to 13.00 MBq (0.03 to 0.35 mCi) [16,17].

### 2.3 RSL step-by-step application

Next, the step-by-step RSL procedure is explained.

- After the surgical referral, the radiologist plans the insertion of the seed, using diagnostic images (mammography, ultrasound);
- The implantation of the seed is performed by the radiologist guided by ultrasound or stereotaxic mammography. In this procedure it is necessary the presence of a radiology technician/technologist for image acquisition and the nurse to perform dressings and measurement of the patient’s vital signs.
- The excision of the seed is performed in the operating room by the surgeon with the aid of an intraoperative gamma radiation detector, usually a gamma probe. In addition to the surgical team, the presence of a radiology technician/technologist is required to perform the radiography of the excised sample, which confirms the removal of the lesion with the radioactive seed(s).
- The sample with the seed(s) is sent to the pathologist, that will perform the removal of the seed and then process the surgical sample in the usual way;
- The removed seed is deposited in a lead box and sent to the radiology sector, where it can be stored for decay and later disposal or returned to the manufacturer, usually supervised by the responsible medical physicist.

This paper presents the steps to implement RSL in a hospital. It contains considerations on:

- Program Implementation Committee
- Licensing
- Professional Team Formation, Education, and Training
- Material and Instrumentation Associated with the Technique
- Inventory for Tracking Seeds
- Radiological Safety Aspects
- Seeds Request and Storage
- Patient Consent
- Radiological Procedure for Placing Breast Localization Device
- Surgical Procedure of a Breast Lesion Excision Identified by the Radiological Device Preoperative Placement
- Specimen Handling in Pathology
- Seeds Deterioration and Disposal
- Procedure Completion and Patient Release

## 3. Results

### 3.1 Program Implementation Committee

Starting an RSL program can be particularly complex as it involves three different departments: radiology, surgery, and pathology, and each department requires its personnel assessment, finances, and resources according to its organizational division [18,19].

Representative leaders from each department should be members of the RSL program implementation committee. Professionals with experience in manual brachytherapy, imaging studies, intraoperative localization, image-guided biopsies, related radiation search using appropriate instrumentation, breast interventionism, and breast-conserving surgery are recommended [20,21,22].

The committee assumes responsibility for evaluating the necessary processes and presenting the schedule for program implementation, supervising the implementation process, and writing the institution-specific standard operating procedure protocol. They are also in charge of making sure that pathology, radiology, and surgery staff who plan to handle radioactive seeds are identified and receive the appropriate instruction [23,24].

### 3.2 Licensing

Although Iodine-125 seed is a sealed source for therapeutic use in brachytherapy, its medical use for intraoperative localization is classified as a nuclear medicine procedure. As this is a radioactive material sealed source, the institution should contact the responsible regulatory agency to acquire the appropriate federal and state licensing documentation for each department that will handle the seed and consult the specific regulations [18,20,21,22,23,25]. If they are imported into the country, additional steps might be necessary.

### 3.3 Professional Team Formation, Education, and Training

The professional team involved in an RSL program is multidisciplinary, as it must include the personnel involved in all seed-handling stages, from request to final disposal. Some specialties are: breast surgeon, radiologist, pathologist, medical physicist, and radiology technologist, plus professionals from related areas such as nurses, pathology assistants, surgical staff, and administrative staff [19,20,22,23].

All professionals must receive training appropriate to their responsibility level. The training can be initiated with lectures and discussions including theoretical aspects of breast-conserving surgery, intraoperative localization, image-guided biopsies, radiological safety, seed handling, and related radiation search using appropriate instrumentation [20].

In sequence, procedure simulation exercises can be developed to practice the seeds insertion and removal, using chicken fillet and inactive seeds (no radioactive material) and later with radioactive seeds. Furthermore, training should include, in addition to routine use, emergency procedures [26].

Each professional must have knowledge and/or training on the procedure development at all stages, be aware of the radiation effects, avoiding personnel contamination and their environment. Radiology technologists can be trained at all stages to follow procedures in the three departments, assisting with imaging and handling of radiation detectors [22,23].

Once the services have started, each team must be supervised by their respective department leader until they complete a minimum number of cases under guidance. The minimum number of cases must be stipulated by the implementation committee, this number must be sufficient to overcome the learning curve inherent to the procedure. After the main team is trained, the additional team members’ training can be accomplished through peer mentoring [18,19].

### 3.4 Material and Instrumentation Associated with the Technique

The equipment includes two Geiger counters for seed documentation in radiology and pathology departments, a navigation system and a portable gamma probe for surgery, and imaging equipment to guide the procedure and for specimen radiography [21,24].

The materials include radiation warning labels that read “caution: radioactive material”, container and vial labels that identify radioactive materials, containers with appropriate protection of lead or equivalent for seed transport, reverse action tweezers, short-term seed storage in radiology, and long-term seed storage in nuclear medicine. In addition to a storage area for the Iodine-125 decay and deterioration with security system [23,26]. It will take up to 2 years for the seed to reach discard radiation level [27].

The cost for the operating room, anesthesia, radiology technicians, and material inventory management is the same for RSL and WGL, not including start-up costs. When the institution already has a nuclear medicine sector, the implementation also becomes more sustainable, as there will be no cost to hire a technical team. In addition, most materials will be already available [19,24].

### 3.5 Inventory for Tracking Seeds

The RSL inventory must be performed, containing information on seed, for example, initial activity, sterilization, storage, implantation, surgical excision, and pathology recovery until final decay in storage or return to the manufacturer. To this end, robust tracking procedures (that trace the seed path inside the hospital) are vitally important to allow ongoing assessment and prevent seed loss. The team professionals should update inventory data at each point of contact throughout the whole RSL process [1,25,26].

The medical physicist is responsible for supervising tacking quality control, use, and proper disposal of seeds. Due to their familiarity with nuclear regulatory compliance, as well as the facilities and procedures for receiving, storing, and decaying radioactive materials, medical physicists with a background in nuclear medicine are valuable individuals in this situation [19,23].

The sequence events in seed tracking include the following departments and activities:

1. Radiology: receiving the seeds; activity verification and inventory register; seed storage in a safe place; seeds transport in a lead envelope to the procedure room; opening the envelope and confirming the seed activity; seed implantation in the breast guided by stereotaxis or ultrasound (more information on these procedures can be found in supporting information).; localization confirmation with mammography and Gamma probe; and registration, in the tracking form, the number of implanted seeds [23,26]. An example of tracking form can be found in supporting information.
2. Surgical center: reception of the patient in the operating room; seed localized in the patient with Gamma probe detector; excised seed and confirmed radioactivity in the surgical specimen with the Gamma probe detector; confirmation of the removal of the seed and lesion with specimen radiography and margins verification; registration in the tracking form, the number of seeds present in the excised specimen [28,29].
3. Pathology: seed localized in the specimen with Gamma probe detector; seed removed from the specimen and deposited in a lead box; register, in the tracking form, the number of seeds removed from the excised specimen [29,30].
4. Radiology: medical physicist collects the seeds in the pathology and forwards them to radiology; seeds are stored for decay and disposal or are returned to the manufacturer; the tracking form is finalized by the medical physicist [21,23].

The seed implantation and surgery dates must be communicated to the radiology department, pathology, and surgery. From the time the radiologist orders the seeds until the seed is returned to the radiology department for disposal, a tracking form must always be started and kept with each seed, with the exception of when the patient is at home. This form must signed by the responsible medical physicist [1].

The tracking form must contain information about patient data, the number of seeds implanted in the breast, and total activity. Additional information should be recorded at various points in the process, such as how many seeds were extracted during surgery, removed from the specimen, and taken by the medical physicist for disposal. The radiologist, surgeon, pathologist, and medical physicist must all date and sign the document. The medical physical professional, responsible for radiation safety in the radiology department, must keep a report of each procedure date and the seed tracking forms [18,19,22]. Furthermore, in supplementary material 1, a template form for tracking seeds is presented.

### 3.6 Radiological Safety Aspects

A radiation safety and security program should include receiving, analyzing, sterilizing, transferring, and storage seeds; safe handling during localization and surgical recovery; and the seed extraction from the specimen by the pathologist [1,20,30]. For this, each department must follow the Standard Operating Procedure protocol established for handling materials during the processes within the program, to avoid the team’s unnecessary exposure.

Professionals should take measures and refrain from handling the seed directly. They should always keep in mind the ALARA principle, an acronym for as low as reasonably achievable. Radiation safety instrumentation must be calibrated and appropriate for the radioactive material types and amounts used in the procedure. Warning signs can be used on procedure room doors while seeds are present with the inscription “Caution: radioactive material” [1,25].

The medical physicist should be responsible for ensuring the quality control and radiological safety program, including staff concerns regarding radiation exposure and seed tracking to avoid potential losses [28].

The protocol should make sure that this is documented and the seed location notified if the surgery is postponed or the patient does not show up for the scheduled surgery. Any loss that requires follow-up should be documented in the patient’s chart, including an absorbed dose estimate [20]. This is because if there is any loss in the custody chain of radioactive materials, regulatory agencies have the power to withdraw or suspend an RSL program’s authorization [19,30].

Each installation’s protocol should include detailed guidelines on what to do in the case of a cut seed incident. Broken or leaking seeds are not expected using typical surgical procedures, so medical staff performing RSL procedures must include safety measures to guard against seed breakage and must be instructed to react in case of a cut or leak [29]. In the unexpected seed leakage case, the medical physics team should be contacted to conduct a radioactive contamination assessment. If the leak is confirmed, all the seed parts must be recovered, and all contaminated materials must be treated as radioactive waste. Thyroid bioassays may be necessary [26].

The professionals who handle seeds or the ones than have the potential to receive more than 10% of the occupational dose limits (1.66 mSv/month [31]) should be monitored by radiation safety procedures [1]. The radiology technologists use a badge monitoring dosimeter. Although minimal exposure to radiation in handling the seed, during insertion, and excision, the radiologist, surgeon, and pathologist should use a badge and ring dosimeters to comply with regulations [25,28].

### 3.7 Seeds Request and Storage

The Iodine-125 seeds must be requested from a supplier licensed to distribute sealed sources for medical use [28] and the material follow pre-approved radioactive material receiving procedures [30]. An activity range level can be requested within each batch to ensure that the greatest number of seeds can still be used during the product’s useful life and last until the next seed shipment arrives [28].

After the receipt, the nuclear medicine team must perform the individual seed assay using a dose calibrator with an appropriate configuration for Iodine-125 low-energy gamma radiation. The values displayed on the radiation dose calibrator must be by approximately 10% of the test declared by the manufacturer [1]. Information about each RSL seed must be entered by the radiation safety team into the RSL inventory record system, including nuclide, the number of seeds, activity, batch number, and seed identification number. The seeds should be stored in a safe place inside a container labeled with the radioactive material symbol and the inscription “Caution: radioactive material” [30].

When necessary to perform the seed sterilization, the nuclear medicine team, behind lead shielding and using tweezers, must place the seeds in individual self-sealed paper packages suitable for sterilization. All packages must then be transported in a sealed container to the sterilization area. Chemical and dry sterilization are not permitted, and temperatures above 138°C should be avoided [22,28].

Both self- and pre-loaded seeds are available. The seed is already put into the needle in pre-loaded seeds, and a rubber stopper holds the stylet firmly in place to prevent early implantation. There are many different needle lengths available, however due to the very limited expiration window for sterilizing needles, it is challenging to supply all lengths. The self-loaded seeds must be manually loaded onto a needle before usage; they are delivered in a sterilized glass container. The radiologist takes up the seed with a tweezer and places it on an 18-gauge spinal needle with bone wax on its tip [22,28,30].

The bone wax prevents the seed from accidental displacement before it is positioned in the center of the target. Too little bone wax can cause the seed inadvertent early implantation in the wrong target, while too much wax can mimic the seed appearance on the ultrasound image or cause misapplication. Is important that the surgeon learn to use both seed types, because of availability issues [1,28].

### 3.8 Patient Consent

Based on the surgical forwarding, the radiologist should discuss the procedure with the patient, explaining the technique, benefits, and risks, as well as potential queries about the radiation dose. The patient must be aware that once the seed has been inserted, she will undergo surgery to have the seed removed. Therefore, sufficient, clear, verbal, written information, and a thorough pre-operative evaluation must be provided to highlight any cause, medical or psychological, that could lead to the surgery cancellation once the seed has been placed [32].

After the suitability for surgery with RSL is confirmed, an informed consent signed by the patient is requested. Furthermore, during the procedure, other data are recorded in the patient’s chart, such as lesion type (nodule, microcalcifications, previously biopsied lesions), localization method (mammography, ultrasound), seed activity and type, insertion and removal dates, radiologist and surgeon, surgical procedure duration, specimen volume, surgical specimen radiographic documentation; and the pathologist’s report [1,32].

### 3.9 Radiological Procedure for Placing Breast Localization Device

The seeds must be transported in a lead box by the radiology technologist in the nuclear medicine sector to the ultrasound imaging room or stereotaxic mammography [1]. Before RSL implantation, the radiologist must ensure that the patient’s prescription and consent are in place. Before the seed is inserted, the patient must confirm the date of the surgery to ensure they can and will return [32]. Allergy review, skin preparation, lesion site identification, and then a local anesthetic administration should be performed [28]. The technique must be performed in a sterile field and the radioactivity seed must be verified before implantation [22,30].

Under imaging guidance, the needle tip is placed in the lesion site. Once it has been directed to the area of the breast lesion, the seed is implanted in the breast parenchyma through bone wax, moving the needle stylet forward [28]. The seed should be positioned in the lesion site center, with a tolerance of ~10 mm [19]. After the needle removal, the seed positioning is verified and documented with mammography without magnification, so that the distances to the nipple and midline are calculated in real size [26].

The second confirmation of the seed implant must then be performed using a gamma detector [1]. Seed activity and reference date can be registered directly on the mammography image, which must be available to the surgeon [25]. In addition, a radiation search should be realized to ensure that no seeds are left on the needle. No one is allowed to leave the room before double confirmation has been completed. No material may be removed from the tray and dumped. If a seed is missing, it must be located. [19,28].

The localization approach is not limited to the surgeon’s incision planning, so the skin entry site does not depend on or dictate the surgical approach, allowing any angle to be use. A needle long enough to reach the target must be used, because it will be inserted through the skin and progressed in any direction that aids in proper localization [26].

Ideally, the seed should be positioned at the lesion edge to minimize the tumor architecture disruption during cutting and the seed recovery in pathology [19]. Placing the seed within the clip bioabsorbable polymer is not ideal as the clip due to the slippery nature of the polymer, it can protrude from the lesion during surgery. [21]. Placing the seed within a collection of fluids, cysts, and bruises is also not ideal as the seed can move freely in the cavity, making placement inaccurate. Furthermore, the seed can be aspirated into the suction container at the surgery time [28].

In cases where the lesion is found deep within a dense breast, the use of seed with higher activity can improve detection [1]. It can be challenging to reach these lesions with standard-length needles when they are in the breast’s central posterior region; a longer needle may be needed. [28]. The vasovagal reaction risk during the procedure is increased with anxiety, reactions range from dizziness to severe bradycardia, so whenever possible, the patient should remain seated during the procedure to increase their comfort [19].

The choice between ultrasound and stereotaxis to guide the localization depends on the original lesion characteristics and the radiologist’s preference. In general, the placement should be performed in the modality in which the lesion is most clearly visible [19]. In the ultrasound room, the radiologist and a nursing assistant will be present, while in the mammography room, in addition to these, the radiology technologist who specializes in mammography will be present.

When possible, ultrasonography guidance is used because it is frequently the patient’s most comfortable localization technique [26]. It is advised to place seeds under ultrasound guidance if the lesion is very superficial in order to increase the distance between the skin entry site and the lesion and reduce the likelihood of poor implantation or extrusion [28].

The seeds are well visualized on mammography, being the best method to verify if the seed has been effectively expelled from the needle. It can be more difficult to confirm seed ejection with ultrasonography since the air or wax plug can introduce doubts. Thus, post-localization mammography is strongly recommended, even in ultrasound-guided localization cases, to document seed implantation as well as ensure proper seed placement for clinical purposes [21,23].

Due to radiological safety and security issues, mammography or ultrasound-guided seed placement are preferable to magnetic resonance imaging. If a seed is lost during the procedure, a search would be impaired due to radiation detection instruments not working normally in the presence of high magnetic field. Overcoming this limitation, an MRI-guided RSL protocol is already available in the literature [33].

If a seed is placed in the incorrect location, either being misplaced or by migrating from the initial implantation site, the seed cannot be repositioned or removed percutaneously. More seeds can be implanted to more precisely locate the lesion site [18]. In these cases, the radiologist should speak with the surgeon to decide whether the lesion is close enough for an exact excision or whether a second more precise locating device needs to be inserted. In the latter scenario, the surgeon can utilize the gamma probe to locate the lost seed after the initial lesion has been removed. This is accomplished through the same incision, with no more breast tissue being removed [19,28].

In the case of multiple or enlarged lesions, the same breast can accommodate numerous seeds. If more than one seed is needed, they should be placed apart from one another far enough to allow for discrimination using a handheld gamma probe, preferably at least 2–3 cm apart [28]. It is recommended to implant a maximum of three seeds in a single breast [22]. In addition, the gamma detector cannot confirm the placement of the additional seeds once the first seed has been placed, so confirmation can only be done through post-localization mammography [25].

At the procedure end, the maximum activity site must be detected with the gamma probe for marking the skin with a tattoo [1]. The technologist should provide the patient with radiation safety instructions, emphasizing the return to surgery [25]. The patient must be given a special identification, an armband, e.g., that alerts others to the radioactive material’s presence.

If the patient is traveling, she should be given a card that states she has radioactivity measurable levels that can be detectable. The planned surgery date must be written on the card. The radioprotection office telephone number of the medical physics department should be provided in case of patient queries [24,25]. Then, the patient will be free to return home, being able to perform together with his family the most usual activities without excessive radiation exposure. There are no additional indications for isotope activity [34].

### 3.10 Surgical Procedure of a Breast Lesion Excision Identified by the Radiological Device Preoperative Placement

On the surgery day, the pre-operative nurse should assist the patient with a chlorhexidine sponge bath or other skin preparation, start intravenous fluids, review the patient’s health history, and confirm the surgical consent form [21]. The surgeon will meet with the patient in the pre-operative area to confirm the seed with a gamma detection placed over the breast [1].

If the sentinel lymph node biopsy (SLNB) is scheduled for the patient, a nuclear medicine team member may come to the preoperative area to inject Technetium-99m [25]. Standard intraoperative gamma probes used for SLNB detection can be used to locate the Iodine-125 seed and guide surgical removal if they can detect low-energy photons (~30 keV). The use of correct probe configurations, energy window, and sensitivity are important to ensure the signal differential detection from the sentinel lymph node and the seed [1,28]. More information on this procedure can be found in supporting information.

Although the Iodine-125 signal is not detected when using the Technetium-99 m setting, some of the Technetium-99 m signal diffusion can be detected with the Iodine-125 setting. But, because the seed is a radioactivity point source, it is sufficiently localized to overcome any contribution from the Technetium-99m [1].

The mastectomy specimen with 1.85 MBq or more of Iodine-125 is sufficient to overcome the Compton effect of the Technetium-99 m standard injection of 16.65 MBq used for SLNB [35]. The Iodine-125 seed should be chosen so that the remaining seed activity proportion and the remaining Technetium-99m activity in MBq are greater than 0.3. Thus, using seeds with at least 1 MBq at the surgery time is enough to achieve sufficient contrast rates considering ~8 MBq of Technetium-99m [36].

Throughout the surgery, the surgeon should evaluate the mammograms that shows the seed implantation. The entry point chosen for localization is not necessary when planning the surgical incision. Additionally, during the process, the gamma probe continuously emits audio input regarding the lesion’s location and distance, enabling continuous real-time reorientation [19,23].

The seeds are titanium coated so the electrical current from the electrosurgical unit will not cause cutting or leakage. The surgeon can safely use electrocautery to dissect the tissue to remove the seed and target area. However, until the seed is isolated and safeguarded, scissors and suction cannot be employed [29]. If suction is required during the procedure before seed removal, a protective stocking can be placed inside the suction container to trap a seed if it is inadvertently aspirated during surgical excision [28].

The breast should be scanned using the gamma probe to locate the seed and determine the incision site [1] so that the lesion is excised with the seed incorporated. Then the gamma probe should be used to confirm that the seed is contained in the resected specimen, and the Iodine-125 activity absence in the breast [28].

The radiology technician should to get a specimen radiography straight after excision to make sure the seed and lesion are inside the specimen [19]. This radiograph confirms that the seed was not misplaced in the operating room and provides as additional confirmation that it was in fact taken from the patient. The pathology team can utilize these radiographic pictures to orient the material, recognize the lesion, evaluate the margins, and find the radioactive seed [29].

While in some cases specimen radiography may be eliminated to reduce operating time, this should be a program implementation committee decision, especially for bracketing, as it serves as a crucial check to make sure the right number of seeds have been removed. In general, specimen radiography is very necessary when there are technical problems in identifying the seed in the specimen when the Iodine-125 seed is not placed exactly in the lesion center, when the surgeon is not sure whether the Iodine-125 seed and the lesion have been removed, when the Iodine-125 seed is displaced from the specimen, when two or more Iodine-125 seeds were used, and/or multiple lesions must be excised [37].

The precautionary procedures are to treat the seed as a lost seed if it is not visualized on the specimen radiography, so the patient and procedure room should then be examined immediately [1,25]. The mastectomy cavity should be checked for seeds using the gamma probe. If seeds are not found, the suction device, suction tube, suction container, surgical sponges, surgical drapes, and even the operating room floor must be scanned. The gamma probe should be used to find the radioactive seed. If the seed is still missing, a radiation security team must search the operating room and any other required areas for it, probably using a more sensitive detector. Nothing and no one must leave the operating room until the seed is found [19,28].

The specimen with the seed should be removed and placed on a specimen plate with an adhesive noting the Iodine-125 isotope presence, the number of seeds, and a “Caution: Radioactive Material” tag, to ensure that all handlers are aware of the radioactive material is present within the specimen [30]. The surgeon can recheck the gamma counts on all six margins to determine distance after removing and orienting the specimen (more information on this procedure can be found in supporting information).

When scheduling the surgery, the pathology team must be informed about the RSL specimen. Additionally, the presence of the radioactive seed must be noted on the pathology request and informed to the pathology laboratory staff upon delivery of the specimen, which must be labeled in accordance with standard pathological procedure, including patient identification and access numbers [30].

### 3.11 Specimen Handling in Pathology

RSL specimens do not require additional safety measures beyond those used for all other specimens in the pathology laboratory [29,30]. An extra patient tag must accompany the specimen to the pathology to follow the seed for storage. Additional labels may be required for intraoperative re-excision and SLNB [28].

The pathology team must recover the seed during the specimen macroscopic examination. Before cutting, the pathologist’s assistant must perform a visual inspection along with a gamma probe to identify the seed location within the specimen, the area with the highest reading must be targeted and sectioned. The tools used for removal must be long-handled, but not perforators, such as forceps [28,29]. After removal, the explanted seeds should be placed in small specimen bags marked with the patient’s name inside a lead-shielded storage container [25].

Maintaining specimen orientation requires attention. This is easily performed using ink and accepted best practices (see supporting information). The remaining mastectomy material must also be examined to determine the presence or absence of seeds after the radiation detector has assessed the tissue macroscopic section. The conventional intraoperative frozen section examination or formalin fixation can be used to continue processing each section that does not produce radiation [30].

The scalpels used in pathology to dissect tissue carry a low risk of the seed being cut or damaged. It is recommended not to dissect with scissors to avoid the seed transaction. Additionally, before the pathologist uses the cryostat or microtome to create incisions in any specimen, it is crucial to make sure that the seeds have been eliminated because these tools have the ability to rupture the seed and perhaps induce the release of the radioactive material contained within [29].

As a positive when compared to WGL, the handling of the breast specimen with RSL allows the specimen to be cut from both sides, in addition, the seed does not cause specimen tearing or distortion [30]. It is useful to roughly section the breast specimen perpendicular to the seed’s long axis to aid identification and retrieval. Specimen radiography can be used to guide where the seed is. If the seed cannot be grossly identified, a sodium iodide detector, gamma probe, or Geiger counter should be present in the pathology laboratory [1].

A final radiation search of specimen material should be performed using the gamma probe to verify the absence of any remaining seeds [1]. Once the seeds have been collected, handling requires general safety procedures. After the radioactive seed has been collected, the tissue section maintenance or paraffin blocks are not required by medical or regulatory standards. The seeds are sent to the institution’s radiology department for proper disposal. The specimen can be then processed in the usual way [28,30].

If the pathologist identifies that the lesion has been incompletely excised, the margin marking kit allows accurate identification for further re-excision. Also, if the pathologist uses specimen radiography to identify that the seed is close to a margin, the surgeon can use the markings to identify the margins that are close to the seed. Markings allow the surgeon to excise additional tissue at the surgery time to reduce the reoperation risk [19,22].

The number of radioactive seeds inserted by radiology and recovered by surgery should match the number of seeds removed in pathology, and both numbers should be recorded on the radioactive seed monitoring form. Radiation safety personnel should be informed if there is a mismatch or if a seed cannot be extracted from the specimen, and quarantine procedures should be put in place until the seed is found [1,25].

### 3.12 Seeds Deterioration and Disposal

Periodically, is necessary to retrieve the collected seeds from the pathology and deliver them to radiology department. Properly trained individual must handle collection and disposal guaranteeing that the correct handling and documentation will be made [25]. The individual seed disposition must be updated in the inventory. In order to ensure that all radioactive seeds used for patients undergoing excision have been returned and are fully accounted for, it is crucial that the recovery department cross-check all the records [1].

The seeds can be kept until collection by the manufacturer for disposal or stored for at least 10 half-lives (≈2 years) for decay and disposal in common waste [30]. In the long-term storage case for decay, after the 10 half-lives, the seeds are examined with an appropriate radiation detection instrument in their most sensitive setting. When seeds are verified to be indistinguishable from background radiation levels, the radioactive markings on the package are removed and material placed in non-radioactive medical waste [1]. With 60 days half-life approximately [38], Iodine-125 seeds take almost two years to decay, which is reasonable, given the seed volume is small.

### 3.13 Procedure Completion and Patient Release

After the surgical procedure is completed and the patient is released from anesthesia, the surgical team transports the patient to the post-anesthesia recuperation room. The radiology technologist must remove any signaling used to identify the radioactive seed presence in the patient after surgery is completed or immediately upon coming to the post-anesthesia care unit. The communication with the post-anesthesia care unit nurse should include specific information about the coloring to map sentinel nodes used, the regional blocks’ presence, motion limitations if the surgeon removed the nodes, and other pertinent information [19,22,28].

## 4. Discussion and Final Considerations

Initially, plan for the time needed to obtain the radiation license for the Iodine-125 seed use. Regular multidisciplinary team meetings during program development are important to create a realistic timeline, having briefing meetings after the first 1-5 RSL cases and having annual or biannual follow-up meetings to discuss any issues or incidents. The implementation committee should conduct a careful initial selection of patients for the procedure, with one or two procedures performed weekly until workflows are established, followed by progressive expansion.

The learning curve for radiologists and surgeons can be short as skills are transferable from the WGL technique. Pathologists must learn new skills and the learning curve is correspondingly longer because is necessary to successfully find the small seed without distorting the specimen.

A single licensed institution is ideal for seed implantation, removal via surgical excision, and disposal. However, if an outpatient surgical center or pathology laboratory is separate from the institution where the seed is implanted, those centers must also have a radioactive materials license authorizing the removal, receipt, transport, and handling of the seed. Seeds can be placed in any properly licensed facility.

It is highly recommended to document patient seed implantation and patient seed removal using post-localization mammography and specimen radiography, respectively. These registers need to be accessible for inspection. The nuclear regulatory license could be in jeopardy and the entire RSL operation may be put on hold if all radioactive seeds are not correctly identified.

Compared with WGL technology, RSL has shown lower rates of positive surgical margins, reoperation, and disease recurrence. Results that suggest that RSL is superior to WGL in terms of surgical efficiency [39].

The longer the interval time between the localization with RSL and the surgery (about 2 months, when with WGL it is a maximum of 1 day) results are significant for the radiology team, due to the ability to perform all week procedures combined. For the surgical team, as the patient undergoing RSL can be the first of the day without delays associated with waiting for the implant to be performed on the same day, is also a major advantage.

Patients who receive RSL prior to neoadjuvant chemotherapy avoid another invasive intraoperative localization device placement procedure prior to surgery. In this scenario, the radioactive seed can be implanted before the neoadjuvant chemotherapy initiation. This represents an increase in safety for patients who achieve a complete pathological response, because the seed will continue to mark the tumor site for further surgery even after the tumor regression.

In the future, we plan to implement this protocol and report the difficulties, solutions found, and adaptations necessary to fit an actual hospital facility and routine.

## Additional File

The additional file for this article can be found as follows:

10.29337/ijsp.182.s1 Supporting information. Radioactive seed tracking form; Location Techniques; Sentinel lymph node biopsy; Image Test – pathology.
