# Supplementary material for: Radioactive Seed Localization for Conservative Surgery of Nonpalpable Breast Cancer: Recommendations for Technology Implantation Program
Source: Int J Surg Protoc. 2022 Nov 15;26(1):94–106. doi: 10.29337/ijsp.182 (PMC9673601; doi:10.29337/ijsp.182)
Supplement: Supporting information. — Radioactive seed tracking form; Location Techniques; Sentinel lymph node biopsy; Image Test – pathology. [file ijsp-26-1-182-s1.pdf]

Supporting information for

Radioactive seed localization for conservative surgery of nonpalpable breast cancer: recommendations for technology implantation program

1. Radioactive seed tracking form

Table 1 presents an example for radioactive seeds tracking form beginning with medical request to the seed's final recovery for disposal.

Table 1 – Radioactive seed tracking form.

THIS FORM MUST ACCOMPANY THE SEED AT ALL MOMENTS
RADIOACTIVE SEED (SEALED SOURCE)<br>REQUEST FOR RADIOACTIVE ISOTOPE ADMINISTRATION AND REGISTRATION
• FILL OUT AT THE REQUEST TIME (Radiologist)<br><br>PATIENT IDENTIFICATION<br>Name: ____ Gender: ___________<br>Birth date: __/__/__<br>Patient identification number: _________________<br><br>LOCALIZATION PROCEDURE:<br>Date: __/__/__ Schedule: _________<br>SURGERY PROCEDURE:<br>Date: __/__/__ Schedule:_________<br><br>RELEVANT CLINICAL INFORMATION<br>Lesions number to be locate:<br>Right breast: ____ Left breast:____<br>Bracketing (multiple seeds)localization is necessary? ____________________________<br>(Multiple lesions and/or bracket lesions >/ 20mm)<br>Image method to guide the localization: _______________________________<br>Needle lenght: ___cm<br><br>ISOTOPE DETAILS<br>Isotope: Iodine-125<br>Seed activity:___MBq<br>Implant type: temporary<br>Total number of seeds for this patient: ______<br>Name: ____________________ Signature:_________________________ Date:__/__/__
• THE NUCLEAR MEDICINE RADIOLOGICAL PROTECTION SUPERVISOR AUTHORIZATION

Note: the isotope cannot be ordered until this signed order is received.
Name: ____________________
Signature:_________________________ Date:__/__/__

- FILL OUT AFTER COMPLETING THE LOCALIZATION PROCEDURE (radiologist)

For confirmation that the seed was implanted as planned.
Date: __/__/__ Schedule:________
Administered by (radiologist): _________________________________
Institution name: ____
Isotope: Iodine-125
Total number of seeds: _____ Total activity:_____MBq
Notes:____
____
Name: ____________________
Signature:_________________________ Date:__/__/__

- FILL OUT AFTER COMPLETING THE SURGICAL PROCEDURE (surgeon)

For confirmation that the isotope was surgically removed as planned.
Date: __/__/__ Schedule: ________
Removed by (surgeon): ____
Institution name: ____
Isotope: Iodine-125
Total number of seeds:_____
Notes:____
____
Name: ____________________
Signature:_________________________ Date:__/__/__

- FILL OUT AFTER RECOVERING THE SEED FROM THE SURGICAL SPECIMEN (pathologist)

For confirmation that the isotope was surgically removed as planned.
Date: __/__/__ Schedule: ________
Recovered by (pathologist): _____________________________________
Institution name: ____
Isotope: Iodine-125
Total number of seeds:_____
Notes:____
____
Name: ____________________
Signature:_________________________ Date:__/__/__

- MEDICAL PHYSICIST ONLY

The medical physicist finalizes the form after collecting the seeds from the pathology sector.
Seeds implanted number: ____
Seeds removed number: ____
Notes:____
____
Name: ____________________
Signature:_________________________ Date:__/__/__

2. Location Techniques [1-3]

Pre-surgical marking is a technique that consists of implanting a marker in the breast lesion to be removed, with the objective of assisting the lesion site with precise identification during the surgical procedure. This marker implant procedure in the breast is guided by an imaging method, which can be an ultrasound or stereotactic mammography.

In the case of digital stereotaxic-guided marking, the breast is positioned on the mammography device, and images are acquired at different angles, usually 0°, -15°, and +15°, for the lesion's precise location using the three-dimensional coordinate system. After local anesthesia, a guide needle is introduced, through which the marker will be implanted, and then the needle is removed. The marker's correct positioning is verified through other mammography images.

In the case of ultrasound-guided marking, the lesion is located, and the needle is introduced with the marker with the assistance of ultrasound images. After the end of the procedure, two mammographic views of the marked breast are performed to verify the final procedure.

3. Sentinel lymph node biopsy [4, 5]

Breast cancer may infiltrate the nearest lymph nodes. To diagnose lymph node staging, a sentinel lymph node biopsy (SLNB) is performed. To perform the SLNB, a radiopharmaceutical is injected into the patient's breast, generally using phytate-Technetium-99m. After a few hours, the nuclear medicine team, using a gamma radiation detector (gamma probe), begins the biopsy procedure, locating the first compromised lymph node. After the lymph node is removed, the specimen is sent to the pathology team for freezing and analysis.

The identification of this first lymph node and its analysis by the pathologist, to assess whether it has the disease, allows the patient to be spared the resection of the remaining axillary lymph nodes when this node is disease-free. Avoiding this resection is important, as it prevents the woman from developing lymphedema, pain, muscle loss, and having an increased risk of infection.

4. Image Test – pathology [6, 7]

Negative surgical margins are the goal of breast cancer-conserving surgery as it refers to the amount of normal breast tissue around the tumor removed during surgery. Tumor-free surgical margins are related to lower rates of reoperation and disease recurrence.

The surgical margins evaluation consists of microscopically analyzing the surgically removed fragment borders, in search of possible neoplastic cells to determine if there are remnants of the neoplasm in the patient or if the lesion was completely removed.

After the surgical procedure, the fragment is fixed for the subsequent cleavage phase. The fragment is then painted with a specific dye to delimit the surgical borders, making the microscopic analysis easier and safer, and then this fragment is sectioned.

Surgical margins free of neoplastic cells are understood to be clean margins, when no cancerous cells are found in the fragment. Narrow margins are those that present neoplastic cells close to the margins, but they are not placed right at the border. Compromised margins are understood to be those that present neoplastic cells on the borders of the surgical piece, indicating that the cancer cells can still be found inside the patient. If, after the margins analysis, one of them is compromised, an intraoperative re-excision should be performed to completely remove the neoplastic cells.

References

1. Taylor DB, Bourke AG, Westcott E, Burrage J, Latham B, Riley P, et al. Radioguided occult lesion localisation using iodine-125 seeds ('ROLLIS') for removal of impalpable breast lesions: First Australian experience. J Med Imaging Radiat Oncol. 2015;59(4):411-20. Epub 20150414. doi: 10.1111/1754-9485.12302. PubMed PMID: 25871837.

2. Bourke AG, Taylor DB, Westcott E, Hobbs M, Saunders C. Iodine-125 seeds to guide removal of impalpable breast lesions: radio-guided occult lesion localization - a pilot study. ANZ J Surg. 2017;87(11):E178-e82. Epub 20160316. doi: 10.1111/ans.13460. PubMed PMID: 26990046.

3. Frost R, Reed AJ, Dessauvagie BF, Taylor DB. Pre-operative localization of impalpable breast lesions using iodine 125 seeds: Placement accuracy and multidisciplinary challenges. Clinical Imaging. 2021;73:124-33. doi: 10.1016/j.clinimag.2020.12.008.

4. Verma S, Taylor D, Al-Ogaili Z. Influence of preoperative breast cancer localization techniques on rates of sentinel lymph node visualization with preoperative lymphoscintigraphy. Nucl Med Commun. 2020;41(9):871-4. doi: 10.1097/mnm.0000000000001245. PubMed PMID: 32796474.

5. Pouw B, van der Ploeg IMC, Muller SH, Valdés Olmos RA, Janssen-Pinkse LK, Oldenburg HSA, et al. Simultaneous use of an 125I-seed to guide tumour excision and 99mTc-nanocolloid for sentinel node biopsy in non-palpable breast-conserving surgery. European Journal of Surgical Oncology (EJSO). 2015;41(1):71-8. doi: https://doi.org/10.1016/j.ejso.2014.10.046.

6. Dessauvagie BF, Frost FA, Sterrett GF, Hardie M, Parry J, Latham B, et al. Handling of radioactive seed localisation breast specimens in the histopathology laboratory: the Western Australian experience. Pathology. 2015;47(1):21-6. doi: https://doi.org/10.1097/PAT.0000000000000197.

7. Cox CE, Furman B, Stowell N, Ebert M, Clark J, Dupont E, et al. Radioactive Seed Localization Breast Biopsy and Lumpectomy: Can Specimen Radiographs Be Eliminated? Annals of Surgical Oncology. 2003;10(9):1039. doi: 10.1245/ASO.2003.03.050.
